# Conformal Pad-Printing Electrically Conductive Composites onto Thermoplastic Hemispheres: Toward Sustainable Fabrication of 3-Cents Volumetric Electrically Small Antennas

**DOI:** 10.1371/journal.pone.0136939

**Published:** 2015-08-28

**Authors:** Haoyi Wu, Sum Wai Chiang, Cheng Yang, Ziyin Lin, Jingping Liu, Kyoung-Sik Moon, Feiyu Kang, Bo Li, Ching Ping Wong

**Affiliations:** 1 Division of Energy and Environment, Graduate School at Shenzhen, Tsinghua University, Xili University Town, Shenzhen City, China; 2 School of Materials Science and Engineering, Georgia Institute of Technology, Atlanta, Georgia, United States of America; 3 State Key Laboratory of New Ceramics and Fine Processing, Department of Materials Science and Engineering, Tsinghua University, Beijing, China; 4 Department of Electronic Engineering, Chinese University of Hong Kong, Shatin, NT, Hong Kong SAR, China; Brandeis University, UNITED STATES

## Abstract

Electrically small antennas (ESAs) are becoming one of the key components in the compact wireless devices for telecommunications, defence, and aerospace systems, especially for the spherical one whose geometric layout is more closely approaching Chu’s limit, thus yielding significant bandwidth improvements relative to the linear and planar counterparts. Yet broad applications of the volumetric ESAs are still hindered since the low cost fabrication has remained a tremendous challenge. Here we report a state-of-the-art technology to transfer electrically conductive composites (ECCs) from a planar mould to a volumetric thermoplastic substrate by using pad-printing technology without pattern distortion, benefit from the excellent properties of the ECCs as well as the printing-calibration method that we developed. The antenna samples prepared in this way meet the stringent requirement of an ESA (ka is as low as 0.32 and the antenna efficiency is as high as 57%), suggesting that volumetric electronic components i.e. the antennas can be produced in such a simple, green, and cost-effective way. This work can be of interest for the development of studies on green and high performance wireless communication devices.

## Introduction

There is an increasing demand for wireless, miniaturized, intelligent and low cost devices in the field of mobile electronics [1–3]. In such devices, one of the largest components is typically the antenna. In order to minimize the size of the antenna, in the last decade, there have been numerous studies of the miniaturization for the antennas; one of the outstanding examples is the design and fabrication of the electrically small antennas (ESAs) [4–6]. As the dimension of an antenna becomes smaller, its bandwidth shrinks and its radiation efficiency is reduced simultaneously. Regarding the operating wavelength, the antenna is electrically small when *ka* < 0.5 (*k* = 2π/λ, *k*: free space wavenumber, *a*: the minimum radius of the sphere that circumscribes the antenna). Those antennas meeting this requirement are called the ESAs [5]. For an ESA, the radiation efficiency, which reflects the antennas’ capability to transmit the input power to radiation power, should be maximized. Additionally, the quality factor (*Q*) should be minimized; *Q* equals to the ratio of stored-to-radiated energy, and is inversely proportional to the antenna bandwidth. However, there exists a physical restriction on the minimum achievable *Q* for a single resonant antenna [5, 7]:

$$Q_{lb}=\eta_{eff}(\frac{1}{ka}+\frac{1}{{\left(ka\right)}^{3}})$$ (1)

where *η*_*eff*_ is the radiation efficiency, and the subscript *lb* denotes the lower bound. Therefore, the ratio *Q*/*Q*_*lb*_ is also commonly used to evaluate the performance of an ESA.

ESAs can be either planar or volumetric. The planar ones are adopted in many applications since their fabrication is simple [8–14]. As compared, the volumetric ESAs usually show superior performance; for example, the improved bandwidth as well as the corresponding quality factor can approach Chu’s limits, whereas their fabrication process is more complicated [4, 15]. Recently, a few research groups have been working on the fabrication of volumetric ESAs in novel manners [16–17]. For example, Best et al. used manually bended wires [4, 18], Adams et al. used a three dimensional (3-D) direct conformal printing through a micronozzle [17] Toriz-Garcia et al. used a 3-D holographic photolithography to fabricate antennas onto a hemi-spherical substrate [19]; Pfeiffer et al. directly transfer-patterned the stamp of the arbitrary patterns onto a contoured substrate through sputtering and then used the electroplating method to obtain the gold based antennas [20]. Jobs et al. pneumatically inflated liquid alloy into a spherical cap [21]. Kim fabricated spherical wire antenna by rapid prototyping conductive layer on a dielectric substrate [22–23]. Previous achievements in this field effectively solved the printing resolution and electrical conductivity problems of the volumetric antennas.

As we recall that Sarma in Massachusetts Institute of Technology first mentioned about the “five-cent tag” (at that time the tags cost more than 50 cents) about a decade ago, the technological advances in the antenna fabrication in the following years have greatly accelerated the application of the radio frequency identification (RFID) tags [24]. The reduced cost greatly changed the logistic & supply-chain industry. Moreover, as suggested by some scientists and sociologists such as Nocera and Whitesides et al. [25–26], the future development of technologies needs to be considered from a cost-conscious aspect. Especially considering that currently 80% of the global population is poor and underprivileged, it becomes more important than ever for them to be benefited from the advances of science and technology, or at least to be provided with better access to valuable information. Low cost wireless electronics may help bring about such a promise to the society, such as to aid those low income people from remote countryside to obtain education and weather forecast information in an affordable manner. Therefore, it is very urgent to develop novel fabrication technologies to produce volumetric ESAs cost-effectively and sustainably, which previous techniques could not make it.

In this work, we demonstrate the feasibility of a simple, cost-effective, and sustainable process for preparing volumetric ESAs by pad-printing method, featured with the extremely low cost and facility requirement with the total production cost potentially lower than 3 US cents, which is comparable to that of the current commercial-available planar antennas. The pad-printing technique adopts an “indirect offset gravure” printing process. This technique is now widely applied in the printing of designed patterns on curved surfaces of toys, keyboards, bottle caps, and potteries etc., making a planar printing available in volumetric requirement, yet there wasn’t report of the involvement of this technique in key electronic component e.g. antenna productions, due to the absence of a suitable ink that simultaneously possesses a considerable conductivity as well as processability, and the lack of technique to solve the pattern distortion problem when transferring the ink. In order to render the electrically conductive composite (ECC, silver paste) compatible to the pad-printing technique, we improved the formulation of the ECC that we developed before [27–28], and we found that batch-fabricating volumetric ESAs by the new method becomes feasible.

**Fig 1:**
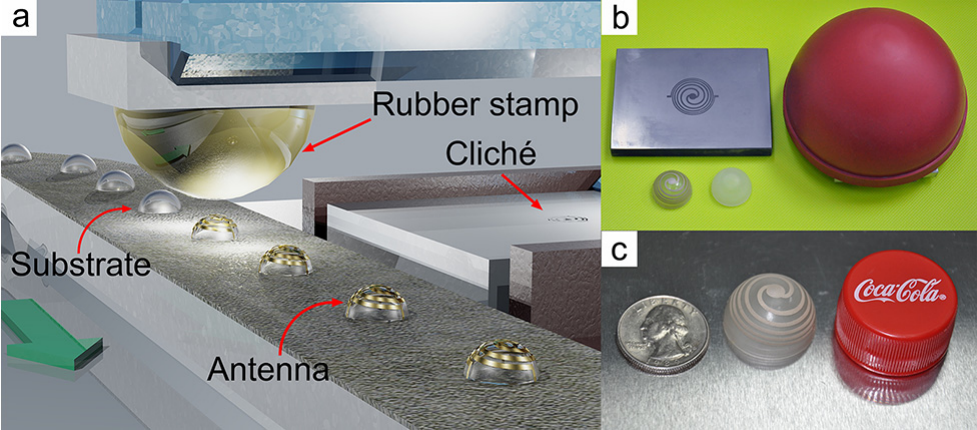
(a) A schematic illustration of the pad-printing process for the ESAs with a spherical helix shape. (b) A photographic image which shows the cliché, the pad, the PMMA substrate and the printed ESA. (c) A photograph of a double helix ESA and a coin.

For demonstration, we choose to fabricate helix antennas on a semi-spherical plastic substrate as the ESAs [18, 20]. In view of the ultralow cost for the packaging and chip price for the commercial-available passive RFID tags, our technological advances may couple with the available chip mounting and packaging techniques and find interesting uses. In order to evaluate the performance of the ESAs made through this process, we designed and fabricated a series of antennas with different layouts on thermoplastic hemisphere substrates; for demonstration, polymethyl methacrylate (PMMA) is chosen as the substrate material, due to its moderate relative dielectric constant (ε_r_ = 3.8 and loss tangent tan (δ) = 0.020, 100kHz) [29]. We found that the antennas fabricated based on our pad-printing method have excellent signal transmittance performance and are much simpler and faster than the ever-reported ones in terms of mass production, which is elucidated in details as follows. As shown in Fig 1, the fabrication process of the ESA samples is quite analogous to printing the plastic cap of a coke bottle, with the estimated production cost of 2.3 US cents. Through a life cycle assessment study, we showed that most of the environmental impact of printing the ESA is at the same level to that of printing the bottle cap for coke (S8 Text), indicative of a green manufacturing characteristic. In other words, pad-printing may be the most suitable one for industrial production of volumetric ESAs.

## Experimental Section

### ECC preparation

Silver micron flakes were purchased from Chengdu Banknote Printing Complex, China. The epoxy resins and hardeners were Epon 828 resin and methyl tetrahydrophthatic anhydride (MTHPA), provided by Shell and Nanya, respectively, and the hexamethylenetetramine used as a catalyst was from Guangzhou Chemical Reagent Factory. Initially, the silver flakes were modified according to literature [27]. The resin and the hardener were mixed according to the ratio of epoxy to hardener which was 1:1 by mole ratio based on the epoxide equivalent weight (EEW) of the epoxy resin and the hydroxyl equivalent weight (HEW) of the hardener. Suitable amount of silver flakes were added to the mixture of resin and hardener. The paste was mixed in a planetary mixer (Hasai Techn. Co., Ltd) in 2,000 rpm for 12 min. After that, a small amount of hexamethylenetetramine catalyst was added to the mixture, accompanied by a moderate mixing process. The electrical resistivity of the ECC was measured by a 4-point probe method (Mitsubishi MCP-T610) after the sample was cured.

### Antennas fabrication

The ESAs were fabricated via pad-printing on a pad-printer (Mini 2S/8, Shenzhen Epole Printing Equipment Co., Ltd.). The cliché, rubber stamp, scraper and roller were loaded onto a pneumatic pad-printer (Feng Sheng Universal Printing Programming System, China). After setting a proper speed (3~5 cm/s) and pressure, the silicone rubber stamp (diameter: 11 cm, height: 8cm) transferred the ECCs from two dimensional antenna patterns to the hemispherical substrate automatically. After curing the samples at 150°C for 15 minutes, the ESAs were obtained. To be noted, the pattern transfer process is accompanied by a nonlinear pattern distortion, which is difficult to predict. The distortion can be corrected in a way demonstrated in S6 Text.

### Antenna measurement

One arm of the antenna was connected to the inner conductor of a coaxial transmission line acting as an antenna feed while the other(s) was (were) secured to a copper ground plane (200 mm × 200 mm). The impedance of the antennas was taken by using a vector network analyzer (E5071C, Agilent Technologies). The impedance characteristics were recorded when the antennas were placed in a free space and in a Wheeler cap respectively. Various size of Wheeler cap was employed to avoid the erroneous measurement in certain frequency ranges. The radiation efficiency of the antennas was estimated using methods from the previous literature [30]. The radiation pattern of the antenna was measured in an anechoic chamber that simulates infinite free space from the Invengo Information Technology Co., Ltd,. The antenna under test (AUT) was placed on a rotating platform in the quiet zone of the chamber. The AUT is then connected to a signal generator (E4438C, Agilent Technologies). A standard gain horn antenna (CBL6112D, TESEQ) was placed at the other end of the chamber and connected to a spectrum analyzer (SMR4503, Schaffner) for measuring the received power. By rotating the AUT and measuring the power received by the horn antenna, the radiation patterns were recorded. The reliability test was carried out by placing the antenna in an 85°C/85RH (relative humidity) conditioning chamber (ESPEC SETH-Z-042L) for 1000 hours, and the electrical resistance and the return loss of the antenna were measured. To quantitatively assess the effectiveness and accuracy of the ECC printing method, both simulation and experimental results were obtained and compared. Computational tool (Ansoft-HFSS) was used to design and simulate the electromagnetic response of the antennas. The model was constructed as an antenna located on a perfect infinite ground plate inside the simulation domain. Signal was fed into the antenna port with proper ground connections to the remaining antenna feeds. Electromagnetic energy irradiated by the system into an air-filled space was then computed. With known simulation parameters appropriate for our cases, e.g. roughness and conductivity conditions, simulated frequency responses of the three types of antennas were obtained. The simulation parameters are summarized as in S9 Text.

### *Q* factor calculation

The fractional bandwidth (FBW) was calculated using the method provided by the previous literature. The half-power bandwidth defined at the -3 dB return loss. The *Q* value is expressed as [4, 17]:

$$Q=\frac{5.828-1}{FBW\sqrt{5.828}}$$ (2)

## Results and Discussion

**Fig 2:**
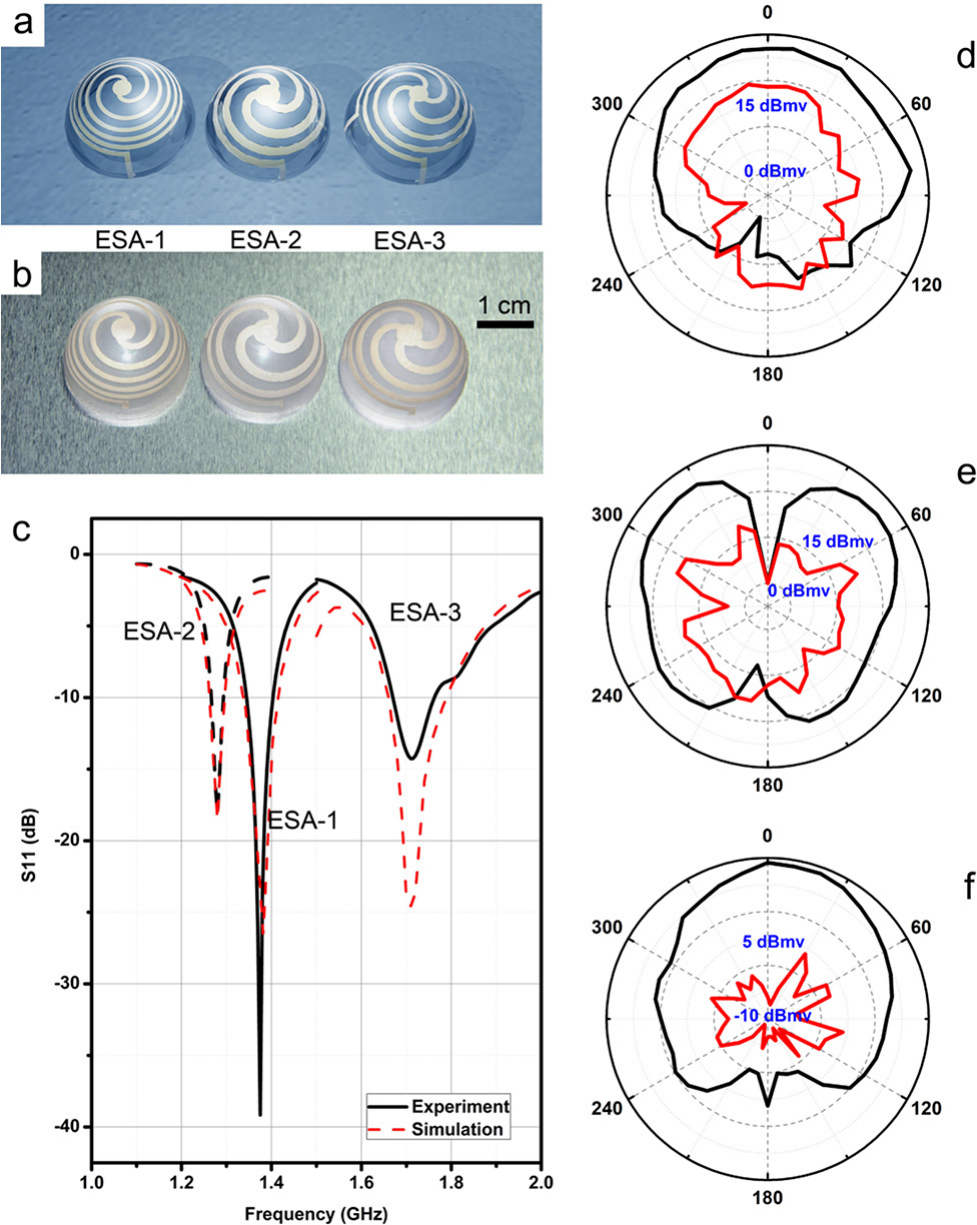
(a) A virtual image of the helix antennas. (b) Photograph of the helix antennas fabricated by pad-printing. (c) Return loss of the helix antennas. (d)~(f) Normalized radiation patterns for ESA samples 1, 2 and 3 (black line: *x-z* plane, red line: *x-y* plane).

As depicted in Fig 1A and 1B, prior to the pad-printing process, a flat pattern of the antenna is designed and engraved on the top of a smooth steel plate, which acts as a cliché (stereotype). We assume that the rubber stamp has a negligible internal stress during printing, and thus the flat cliché pattern can be regarded as a 2-D projection of the intended 3-D antenna pattern. For the printing process, the patterned side of the plate is dispensed with the silver paste, which spreads out homogeneously by rolling. Herein the ECC used to fabricate the antenna contains 66.7 wt% of the modified silver content (resistivity: ~ 8×10^−6^ Ω·cm), for the purpose of an optimal viscosity and a low cost [27]. As the patterned groove is filled, a stainless steel scraper is employed to scrape out all excess silver paste except those filled in the patterned groove. Then a rubber stamp is pressed down vertically onto the plate and touches the silver paste. The rubber stamp has a round shape and the centre is accurately aligned with the centre of the pattern before printing. As the stamp presses down, the air on top of plate is squeezed out, giving rise to a good contact between the ECC and the stamp so that the patterned ECC can be picked up from the groove as the stamp lifts away. After that, the stamp with the ECC is moved and placed above the top centre of a hemi-spherical PMMA substrate (the outer diameter is 24.0 mm and the thickness is 1.0 mm), and pressed against the substrate to transfer the ECC pattern onto the PMMA substrate. Finally, the transferred ECC is cured at 150°C for 15 min in an oven and the antenna pattern is completely fabricated and ready for testing (S1 Text). Inspired by the work of Best et al., we designed and fabricated the spherical helix antennas as the prototype based on our simulations, since they closely approach the fundamental limit of small antennas [18]. This has been confirmed by Kim and Pfeiffer *et al*. [20, 22]. By varying the numbers and turns of each arm, the antennas can be impedance-matched to 50 Ω. Three kinds of antennas with different designs were fabricated with two, three and four radial arms and 2.6, 1.1 and 0.9 turns counter clock wisely, denoted as ESA 1, 2 and 3 respectively (Fig 2A). The widths of the arms varied between 1 to 2 mm (S6 Text). The thickness of the printed arms can be controlled by the pad-printing condition. By a morphology measurement, it was observed that the thickness of the arms was around 20 μm and the surface roughness of the printed area could be as low as 1 μm. To be noted, considering the skin effect of the alternating electric current [31], the skin depth for the ECC based antenna with 1 ~ 2 GHz operating frequency is 3 ~ 4 μm; and thus the arm thickness of 20 μm is able to prevent the radiation efficiency from additional substrate loss, which is caused by skin effect of the ECC. Therefore, the loss of the radiation efficiency of the substrate can be reduced. Details are summarized in S1–S3 Texts.

We observed that the frequency responses from both computational and experimental results were consistent, as shown in Fig 2. The as-obtained antennas were characterized with their signal radiation properties. The results suggest that the sample ESA-1 operates at 1.38 GHz, which is in agreement with the simulation result. Since the diameter of the substrate is 24.0 mm, the *ka* value of this antenna is calculated to be 0.35. The centered operating frequencies of ESA-2 and ESA-3 are found to locate at 1.28 GHz and 1.71 GHz, corresponding to the *ka* values of 0.32 and 0.43, respectively. All of the *ka* values are smaller than 0.5, confirming the electrically small characteristic. The return loss shown in Fig 2C qualifies how well an antenna matches the source. The impedance is matched to 50 Ω, and the return losses of -39, -18 and -15 dB are observed for ESA-1, 2 and 3, respectively, indicating the well-match for the antenna to the sources. At different resonance frequencies, all three types of antennas showed a good frequency-matching with the simulated results, which strongly evidenced a successful conformal pattern transfer.

In addition to the coupling status, the Q factor and radiation efficiency are also the important figures of merit to evaluate the antennas’ performance. The Q factor, defined as the ratio of energy stored to energy radiated, is inversely proportional to the bandwidth of the antenna [20]. Herein, we measured the half-power bandwidth (responsible for the bandwidth at -3 dB return loss) of the antennas, and measured the radiation efficiency, which reflects the ratio of the radiated power to the total accepted power. The radiation efficiency has impact on bandwidth, range and fundamental limits of antennas, and was measured via the Wheeler Cap method [30]. The performance characteristics of each antenna are summarized in Table 1. The half-power bandwidths for ESA-1, 2 and 3 are 13.1%, 7.1% and 23.1%, and the corresponding Q factors are 15.3, 28.2 and 8.7, respectively. The antenna radiation efficiency was measured to be 51%, 57% and 55% respectively, which is comparable to the previous volumetric antennas fabricated by the conformal silver ink printing [17], direct transfer printing [20], liquid alloy inflating^21^ and holographic photolithography [19]. This efficiency is reasonable for its size, and the loss may be caused by the resistivity of the ECC, which is about five times higher than the copper. The enhancement of efficiency can be realized by reducing the resistivity of ECC via a high silver loading. The resulting Q factors are correspondingly 1.2 × *Q*_lb_, 1.5 × *Q*_lb_ and 1.1 × *Q*_lb_ respectively, which means that our antennas can meet the fundamental limit of ESA. For Fig 2D, the sample ESA-1 was placed on a horizontal copper ground plane with the apex pointing vertically upward. The horn antenna was placed on the other side of the chamber and the center is placed on the same height of ESA-1 sample. By rotating ESA-1 carefully along its central axis, we were able to record the black curve as shown in Fig 2D. By rotating the sample along the horizontal direction for 90°, the central axis of ESA-1 was aligned with the horn antenna [17]. Then ESA-1 and the copper ground plane was rotated along the vertical axis (same direction in the upper experimental condition), we recorded the red curve as shown in Fig 2D. Sample ESA-2 and ESA-3 were measured in the same condition and the results are shown in Fig 2E and 2F. In summary, the helix antennas fabricated by pad-printing ECC on a hemispherical dielectric substrate can well-couple with the source with both excellent efficiency and a wide bandwidth. The above properties of the pad-printed ESAs are comparable to those reported previously [21, 17, 19, 20].

**Table 1:** Performance of the antenna fabricated by various methods.

	Samples	*ka*	Center frequency (GHz)	Bandwidth (%)	Efficiency (%)	*Q*	*Q/Q*_*lb*_
ECC pad-printing (this work)	ESA-1	0.35	1.38	13.1	51	15.3	1.2
	ESA-2	0.32	1.28	7.1	57	28.2	1.5
	ESA-2R*	0.32	1.28	5.7	62	35.1	1.7
	ESA-3	0.43	1.71	23.1	55	8.7	1.1
Silver ink printing [17]	—	0.46	1.70	15.2	71	13.1	1.5
	—	0.21	0.79	6.3	14	31.9	2.0
Direct transfer printing [20]	—	0.23	1.12	2.0	52	—	2.1
	—	0.31	1.52	5.3	69	—	1.8
Liquid alloy inflating [21]	—	0.22	0.426	1.9	55	53	1.0
Holographic photolithography [19]	—	0.43	2.06	13.6	46	14.7	2.15

* ESA-2R: Sample ESA-2 after the 1000 hours-reliability test.

**Fig 3:**
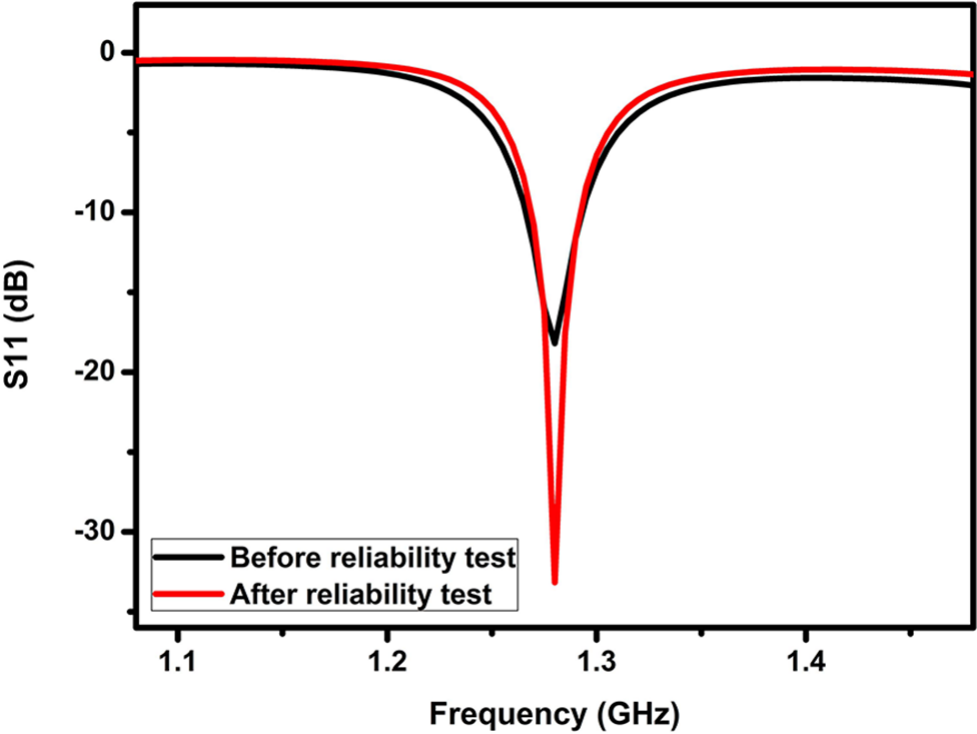
Return loss of ESA-2 before and after the reliability test (85°C/85%RH for 1000 hours).

In order to evaluate the reliability of these ESAs, the sample was conditioned in a temperature-humidity chamber (85°C/85% relative humidity) for 1000 hours and the resistance was recorded. As presented in section 4 in Sopproting Information, the resistance of the antenna falls down to 83% after the aging test, indicating the resistivity of 6×10^−6^ Ω·cm. This slightly reduced resistivity may stem from the further crosslinking of the epoxy binder, which is a common phenomenon, and the signal transmittance performance of the ESA-2 (also denoted as ESA-2R) was maintained after the reliability test [32]. As can be seen in Fig 3, there is a slight shift of the operating frequency of the antenna. The bandwidth is reduced from 7.1% to 5.7%, corresponding to a *Q* factor of 35.1. The radiation efficiency and the *Q/Q*_*lb*_ value can be maintained to be 62% and 1.7 respectively. This increased efficiency is caused by the reduced resistivity of the ECC after the reliability test that results in a narrower bandwidth (5.7%) as well as a higher *Q* factor (35.1). These results show that the signal transmittance performance of the antenna can be well preserved after the reliability test.

Using the pad-printing method for preparing the volumetric ESAs, the production speed can be quite fast, and reproducibility is good. (More than 10 pieces of antennas can be prepared within one minute on the machine that we used.) Moreover, the pad-printer is cheap in price (same level to a planar screen printer). Therefore, when considering at a mass-production level, using the ECC and the pad-printing method is feasible and cost-effective, and the production cost is primarily due to the materials cost. An ECC based electrically small antenna with the efficiency of 50 ~ 60% has the diameter of 24 mm; considering that the weight of the plastic substrate materials and the silver powder, the overall materials cost is estimated to be about 2.3 US cents, (S7 Text) which will be quite competitive in the passive RFID antenna market [24]. Moreover, we note that the materials cost of an ESA is primarily determined by the size of the antenna, especially related to the use of silver, which takes over around 50% of the materials cost. As compared to the current commercial planar RFID antennas which are fabricated by punching, screen-printing or etching, the materials and fabrication cost of our ESAs are at a comparable level. Moreover, the production efficiency is quite high as well, due to the simplicity and extensive applications of the pad-printing technique in the manufacturing industry.

Since the pad-printing technology is quite mature and widely used, the preparation process of the ESAs is analogous to that of a coke bottle cap (as shown in Fig 1C). When being considered from a life cycle assessment point of view, as indicated by Table 2, the environmental impacts of producing such an antenna are comparable to that of producing the cap of a bottle of Coke (S8 Text). Therefore, this pad-printing technique represents both low cost and environmental-benign characteristics.

**Table 2:** Standardized life cycle impact assessment (LCIA) results of a bottle cap and an pad-printed antenna. (The dimensionless numbers are the ratio of the emission equivalent weight to reference value.)

Impact Category	Bottle Cap	ESA
GWP (Global Warming Potential)	1.45 × 10^−12^	3.34 × 10^−12^
POCP (Photochem. Ozone Creation Potentia)	1.26 × 10^−12^	3.74 × 10^−12^

## Concluding Remarks and Outlook

In summary, we for the first time demonstrated the feasibility of transferring the two-dimensional antenna patterns to a three-dimensional substrate surface via pad-printing technique, and the ESAs fabricated by this simple technique showed a radiation efficiency range of 51 ~ 57%, the *Q* factors of 15.3, 28.2 and 35.1, all of which satisfied the fundamental limit of ESA. By designing the appropriate ECC ink formulation and the distortion correction strategy, pad-printing technique, which has been used for the low-end printing purposes, is now ready for printing volumetric ESAs. This technological advance can offer attractive advantages for meeting the demands of more compact size, lower fabrication and environmental budgets, and better control of the printing process. The estimated materials cost of the antenna fabricated by our method is about 2.3 US cents; and the environmental impacts are comparable to manufacturing a coke bottle cap (S8 Text). Concerning the low cost and the other figures of merit, this technique may eventually help achieve sustainably fabricating commercial volumetric antenna tags with the cost within 10 US cents in the near future. We envisage that this scalable, green, and cost effective fabrication technology of the volumetric antennas would arouse great interests to both science community and the industrial sector; we look forward to the broad application of this technique in RFID techniques and wearable electronics etc.

## Supporting Information

S1 Text Materials Characterizations. (DOC)

S2 Text Viscosity measurement of the ECC paste. (DOC)

S3 Text Calculation about the skin depth. (DOC)

S4 Text Reliability test of the ECC antenna. (DOC)

S5 Text Reproducibility. (DOC)

S6 Text Pattern transfer distortion during pad-printing. (DOC)

S7 Text Cost estimation of the ESAs in a mass production scheme. (DOC)

S8 Text Life cycle assessment. (DOC)

S9 Text Parameters used in antenna simulation. (DOC)
