# Supplementary material for: Conformal Pad-Printing Electrically Conductive Composites onto Thermoplastic Hemispheres: Toward Sustainable Fabrication of 3-Cents Volumetric Electrically Small Antennas
Source: PLoS One. 2015 Aug 28;10(8):e0136939. doi: 10.1371/journal.pone.0136939 (PMC4552618; doi:10.1371/journal.pone.0136939)
Supplement: S1 Text — (DOC) [file pone.0136939.s001.doc]

**S1 Text. Materials Characterizations.**


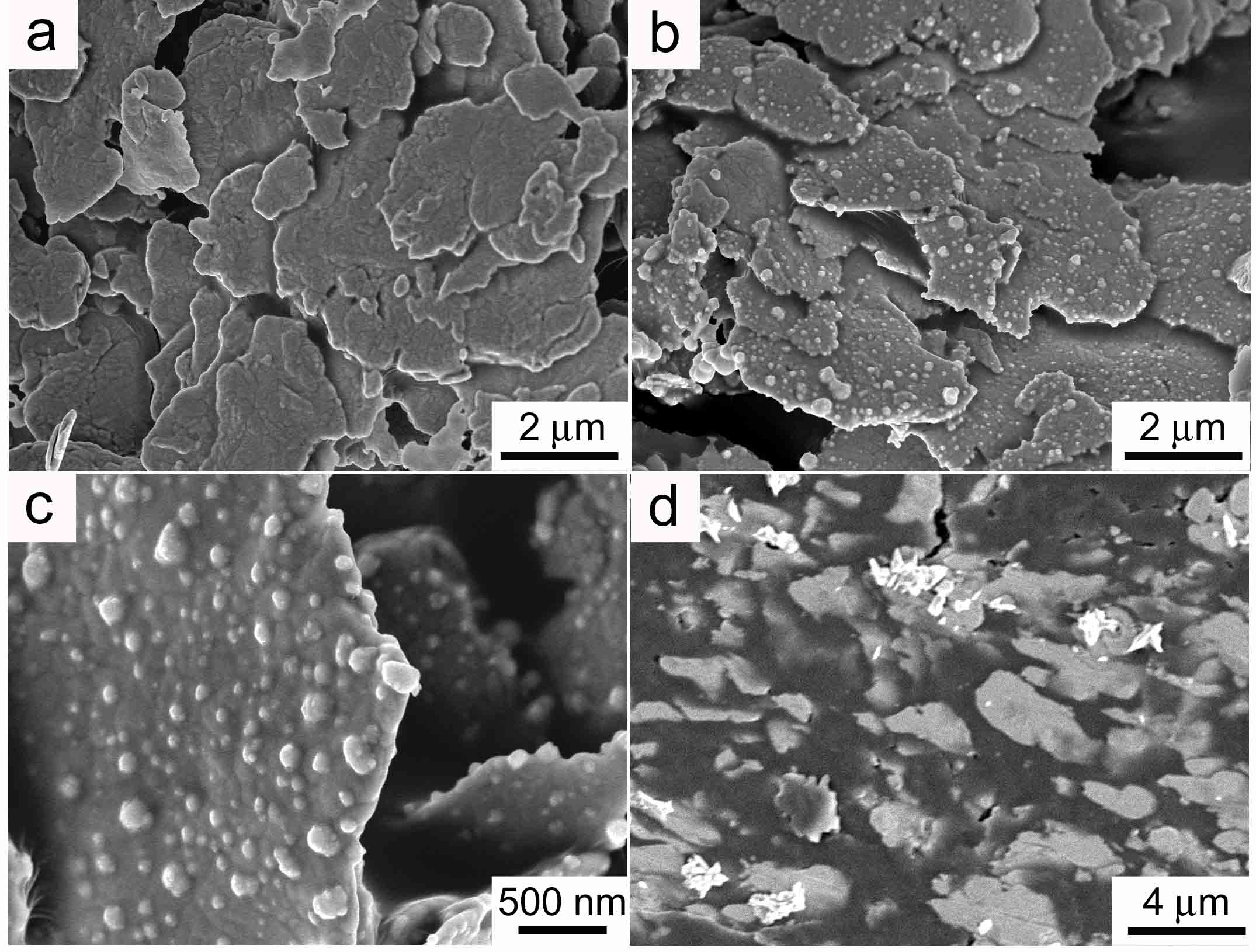


S1 Fig.. SEM images of silver flakes. (a) Bare silver flakes. (b) Surface modified silver flakes. (c) Surface modified silver flakes (high magnification). (d) SEM image of the cross-section of the pad-printed ECC.

In order to obtain an appropriate rheological characteristic to cater to the pad-printing process, the silver content of the ECC is adjusted to about 66.7wt% in a bisphenol-A type of epoxy resin. As investigated in our previous research, surface iodination of the silver flakes can substantially enhance the electrical conductivity of the ECC. A high conductivity is advantageous to the performance of the antennas. Therefore, surface iodination of the silver flakes is adopted and the volume resistivity of the ECC is 8×10^-6^ Ω·cm. The FE-SEM (field emission scanning electron microscopy, Machine: Hitachi S4800) images of the silver flakes are shown in S1 Fig. Prior to iodination, the silver flakes exhibit a smooth surface, whereas small nano-islands appear after iodination, in agreement with previous reports.^[1, 2]^ The cross-section of the pad-printed ECC indicates a homogeneous distribution of the silver flakes, suggesting a consistent conductivity. The surface roughness of the pad-printed ECC was investigated by an Olympus OSL4000 confocal microscope and the estimated roughness is around 0.6 μm, which are demonstrated by S2 Fig and S1 Table. The thickness of the pad-printed ECC is around 20 μm, which is demonstrated by the cross-section of the SEM image in S2 Fig b.


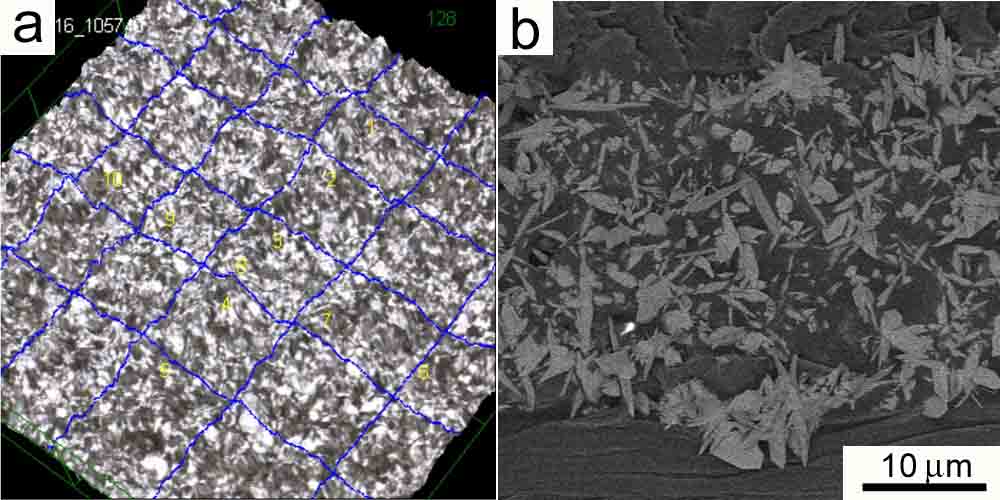


S2 Fig. (a) 3D optical photography of the ECC surface after being pad-printed, indicating the roughness of around 0.6 μm. (Magnification: 1000x) (b) Cross-section SEM image of the ECC by pad printing, indicating the thickness of about 20 μm in this area.

S1 Table. Surface roughness of the ECC after pad-printing.

| Locations | 1 | 2 | 3 | 4 | 5 | 6 | 7 | 8 | 9 | 10 | Average |
| --- | --- | --- | --- | --- | --- | --- | --- | --- | --- | --- | --- |
| Roughness (μm) | 0.464 | 0.578 | 0.702 | 0.759 | 0.650 | 0.650 | 0.650 | 0.650 | 0.650 | 0.650 | 0.650 |

**References**

[1] Yang C, Xie YT, Yuen MMF, Xu B, Gao B, Xiong XM, et al. Silver Surface Iodination for Enhancing the Conductivity of Conductive Composites. *Adv. Funct. Mater.* **2010**; 20(16): 2580-2587. doi: 10.1002/adfm.201000673.

[2] Yang C, Xie YT, Yuen MMF, Xiong X, Wong CP. A Facile Chemical Approach for Preparing a SERS Active Silver Substrate. *Phys. Chem. Chem. Phys.* **2010**; 12(43): 14459-14461. doi: 10.1039/C0CP00414F.
