# Supplementary material for: Conformal Pad-Printing Electrically Conductive Composites onto Thermoplastic Hemispheres: Toward Sustainable Fabrication of 3-Cents Volumetric Electrically Small Antennas
Source: PLoS One. 2015 Aug 28;10(8):e0136939. doi: 10.1371/journal.pone.0136939 (PMC4552618; doi:10.1371/journal.pone.0136939)
Supplement: S2 Text — (DOC) [file pone.0136939.s002.doc]

**S2 Text. Viscosity measurement of the ECC paste.**

The viscosity of such an ECC was measured by an Anton Paar MCR 302 Rheometer (aluminum parallel plates, diameter: 25 mm, plate distance: 0.3 mm) and the result is shown in S3 Fig. The viscosity of the ECC drops substantially with increasing shear rate, which indicates that the ECC is a non-Newtonian shear thinning fluid, and the paste at higher shear rate could reduce the viscosity to provide better processability. When contacting with substrate, the rubber stamp has a translational speed of around 0.5m/s, which corresponds to a dynamic viscosity of 5-10 Pa·s.

A high silver loading is advantageous for enhancing the conductivity of the ECC. However, with the increase of the silver concentration, the viscosity of the ECC increases accordingly. In the same way, too low viscosity may result in the low electrical conductivity and printing difficulties. The silver loading of ECC in our case was adjusted to be 66.7 wt%, which is optimal for transferring the silver paste from the cliché to the hemispherical PMMA substrate.


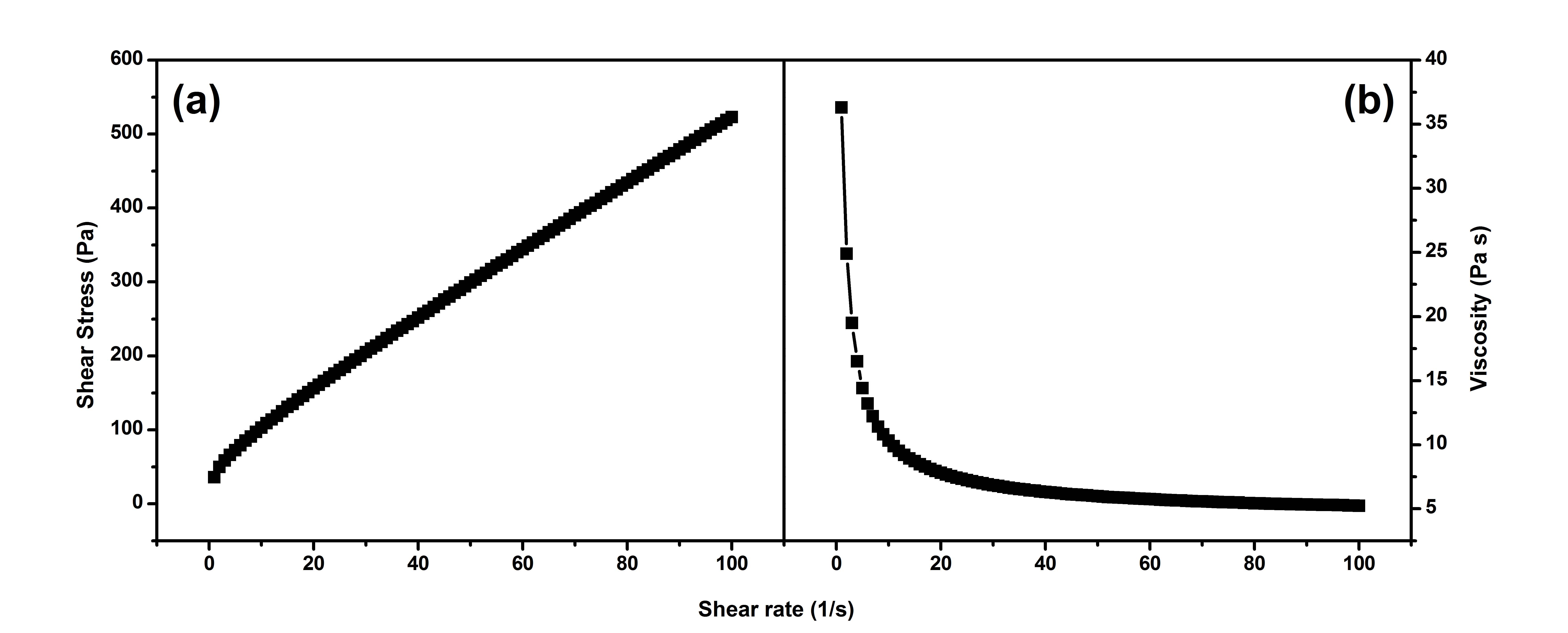


S3 Fig. (a) Shear stress vs. shear rate and (b) Dynamic viscosity vs. shear rate of the silver paste employed in antenna fabrication.
