# Supplementary material for: Conformal Pad-Printing Electrically Conductive Composites onto Thermoplastic Hemispheres: Toward Sustainable Fabrication of 3-Cents Volumetric Electrically Small Antennas
Source: PLoS One. 2015 Aug 28;10(8):e0136939. doi: 10.1371/journal.pone.0136939 (PMC4552618; doi:10.1371/journal.pone.0136939)
Supplement: S3 Text — (DOC) [file pone.0136939.s003.doc]

**S3 Text. Calculation about the skin depth.**

In order to maximize the electrically small antenna (ESA) efficiency, the thickness of the conductor (ECC) should be at least 3δ_s_, where δ_s_ is the skin depth at the operating frequency, and it is calculated as follows [1, 2]:

$\delta_{\text{s}}=\sqrt{\frac{\rho}{\pi f\mu_{0}\mu_{r}}}$ (S1)

where *ρ* is the resistivity of the conductor, *f* is the operating frequency of the antenna, *μ*_0_ is the permeability of free space, which is around 1.257×10^-6^ H·m^-1^, and *μ*_r_ is the relative permeability. Herein, *μ*_r_ is 1 for silver based ECC. The estimated skin depth for ESA-1, 2 and 3 are 3.83, 3.98 and 3.44 μm. In our experimental observations, the thickness of the ECC is about 20 μm (S2 Fig b), which is more than 3δ_s_ for all the antennas. This means that the substrate loss of radiation efficiency can be reduced effectively because of the adequate thickness of the conductive arms.

**References**

[1] Chung DDL. Electromagnetic Interference Shielding Effectiveness of Carbon Materials. *Carbon* **2001**; 39(2): 279-285. doi:10.1016/S0008-6223(00)00184-6.

[2] Brewer SH, Franzen S. Optical Properties of Indium Tin Oxide and Fluorine-doped Tin Oxide Surfaces: Correlation of Reflectivity, Skin Depth, and Plasmon Frequency with Conductivity. *J. Alloy.Compd.* **2002**; 338(1-2): 73-79. doi:10.1016/S0925-8388(02)00217-7.
