# Supplementary material for: Conformal Pad-Printing Electrically Conductive Composites onto Thermoplastic Hemispheres: Toward Sustainable Fabrication of 3-Cents Volumetric Electrically Small Antennas
Source: PLoS One. 2015 Aug 28;10(8):e0136939. doi: 10.1371/journal.pone.0136939 (PMC4552618; doi:10.1371/journal.pone.0136939)
Supplement: S4 Text — (DOC) [file pone.0136939.s004.doc]

**S4 Text. Reliability test of the ECC antenna.**

The reliability of the ECC is evaluated by measuring the resistivity variation during the aging test. The antenna was placed in a conditioning chamber which maintained as 85^o^C/85%RH (relative humidity). After 1000 hours, the resistivity of the ECC reduced to about 6×10^-6^ Ω·cm, which was about 83% of the original resistivity. This enhanced conductivity results in the better coupling of the antenna and source, indicating by the greater return loss. Moreover, the radiation efficiency of the antenna is also enhanced slightly due to the enhanced conductivity.
