# Supplementary material for: Conformal Pad-Printing Electrically Conductive Composites onto Thermoplastic Hemispheres: Toward Sustainable Fabrication of 3-Cents Volumetric Electrically Small Antennas
Source: PLoS One. 2015 Aug 28;10(8):e0136939. doi: 10.1371/journal.pone.0136939 (PMC4552618; doi:10.1371/journal.pone.0136939)
Supplement: S5 Text — (DOC) [file pone.0136939.s005.doc]

**S5 Text. Reproducibility.**

In order to demonstrate the reproducibility of pad-printing, three more 3-arm helix antenna, in addition to ESA-2 were fabricated; their performances are shown in S4 Fig and S2 Table. The operating frequency of these antennas varied from 1.26 to 1.29, and the radiation efficiency is maintained at above 56%, indicating the robustness and reproducibility of the antennas fabricated by pad-printing. The variation of the antenna performance may be related to the minor geometrical variances of each of the PMMA substrate and the alignment errors during pad-printing. Although the glass transition temperature of PMMA is about 105^o^C, we didn’t observe any significant shape change of the hemispheres after curing them in an oven at 150^o^C for a short period of 15 minutes. Meanwhile, according to the antenna characterizations, all antenna samples exhibited excellent signal transmitting performance.


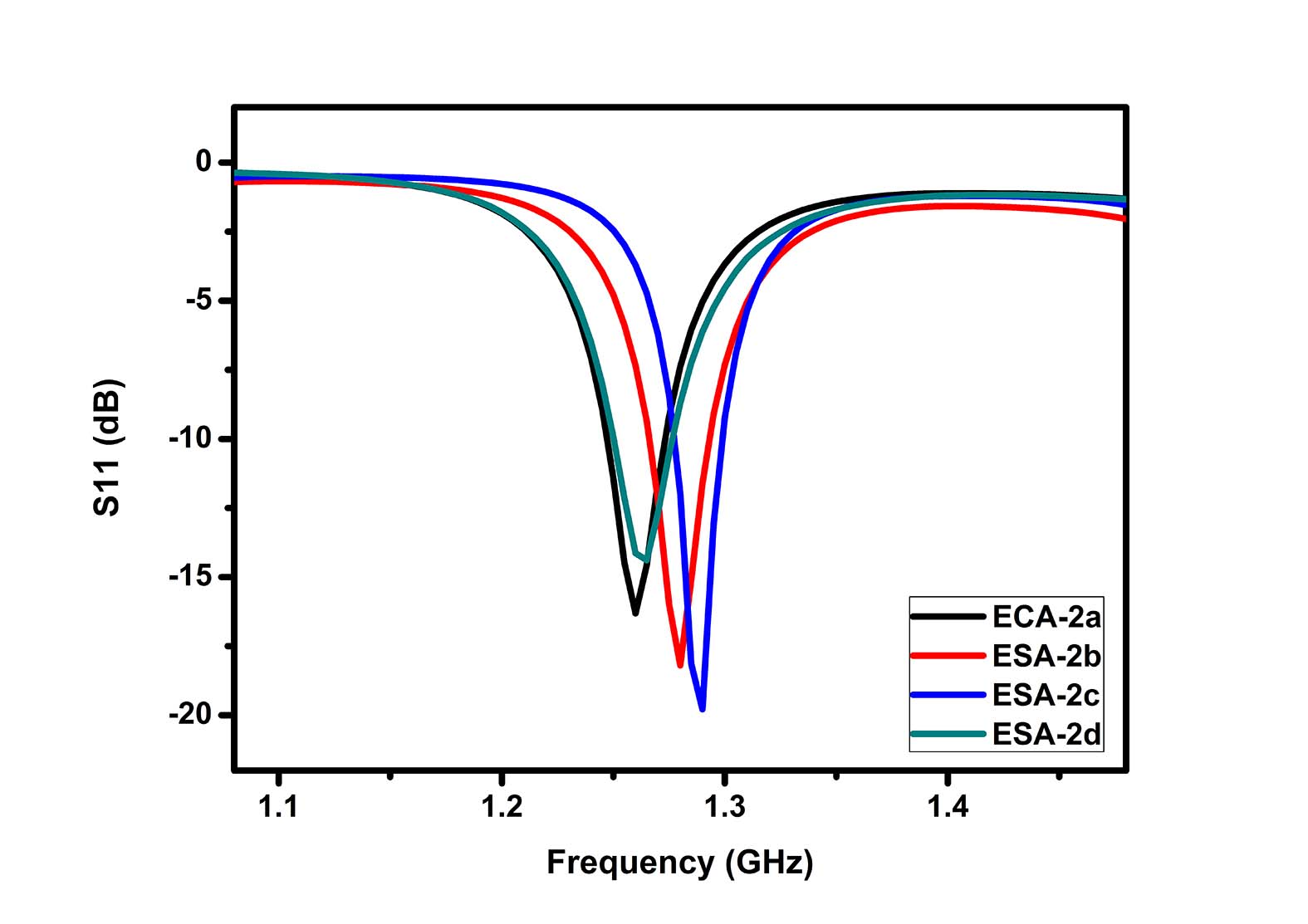


S4 Fig. Return loss of four ESA-2 antennas fabricated the same method

S2 Table. Performance characteristics of four ESA-2 antennas fabricated in the same method

| Samples | *ka* | Center frequency (GHz) | Bandwidth (%) | Efficiency (%) | Q | Q/Q*_lb_* |
| --- | --- | --- | --- | --- | --- | --- |
| ESA-2a | 0.32 | 1.26 | 7.0 % | 60 % | 28.6 | 1.4 |
| ESA-2b | 0.32 | 1.28 | 7.1 % | 57 % | 28.2 | 1.5 |
| ESA-2c | 0.32 | 1.29 | 5.4 % | 62 % | 37.0 | 1.8 |
| ESA-2d | 0.32 | 1.26 | 7.7 % | 56 % | 26.0 | 1.4 |
