# Supplementary material for: Conformal Pad-Printing Electrically Conductive Composites onto Thermoplastic Hemispheres: Toward Sustainable Fabrication of 3-Cents Volumetric Electrically Small Antennas
Source: PLoS One. 2015 Aug 28;10(8):e0136939. doi: 10.1371/journal.pone.0136939 (PMC4552618; doi:10.1371/journal.pone.0136939)
Supplement: S6 Text — (DOC) [file pone.0136939.s006.doc]

**S6 Text. Pattern transfer distortion during pad-printing.**


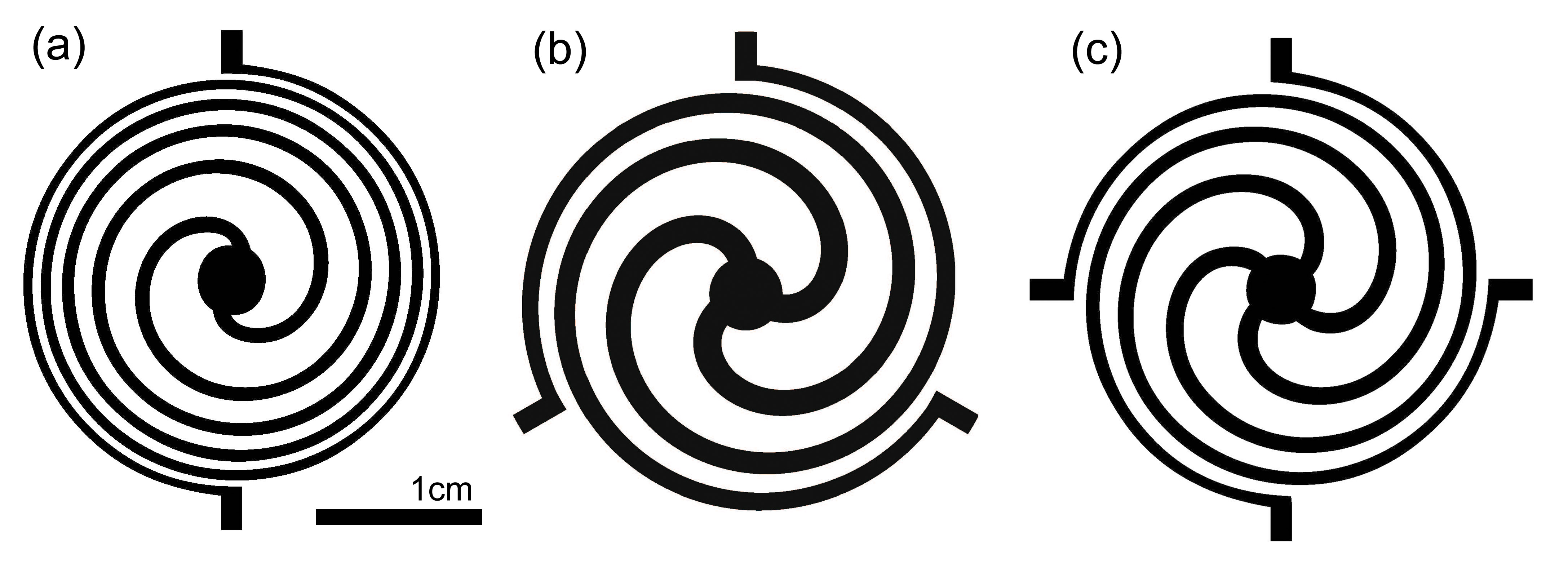


S5 Fig. The geometrical projections of the three antennas on a plane, which were used for fabrication of the cliché. (a) ESA-1 (b) ESA-2 (c) ESA-3.

During the pad-printing pattern transfer, a direct 2-D projection of the 3-D antenna pattern onto the cliché was used (as shown in S5 Fig), which was the case for a rubber stamp with vanishing elastic modulus. The real rubber stamp having finite modulus may lead to pattern transfer distortion. For the above three patterns, this distortion was not considered in this paper, since we found that the antenna signal transmittance performance matched very well with the simulated results without considering this distortion.

Actually, this distortion can be corrected in a simple way. The projected cliché pattern and the rubber stamp can be adjusted as follows. Given particular substrate geometry, pattern design on substrate, rubber stamp shape and mechanical properties, etc., the transformational adjustment can be determined. In our case, the rubber stamp and substrate centers were perfectly aligned, and thus the geometries were axis-symmetric. Therefore, only distortion along radial direction was expected. A concentric circular test pattern with known dimension was designed and transferred to the cliché for printing. The printed test pattern on the hemispheric substrate was then optically captured as digital image and then compared with the perfect concentric pattern to measure the distortion.

The comparison provides us with information of the radial distortion at different position away from the center of the pattern. As shown in S6 Fig a positive distortion means translational distortion away from the center, and a negative distortion means that in reverse direction. The pattern distortion was observed to be between 0.0-0.4mm within 9 mm distance from the pattern center. A higher distortion was observed on the outer-rim (a distortion of 0.4-1.0 mm in the distance of 9 to 12 mm from the center). This distortion effect can be corrected by constructing a linear inverse-transformation on the pattern based on the distortion measurement, which can help to improve the electromagnetic properties of the antenna. In this way, a perfect pattern without printing distortion can be obtained after several iterations. Additionally, the choices of the substrate geometry, rubber stamp's material, rubber stamp's shape and size, and the complexity of the printed pattern are all form factors for this distortion effects. In our experiment, we discovered that this distortion had little effect on the antenna's electromagnetic performance and such pattern correction was not performed. This pattern distortion effect on antenna properties is an interesting topic to be studied in future.


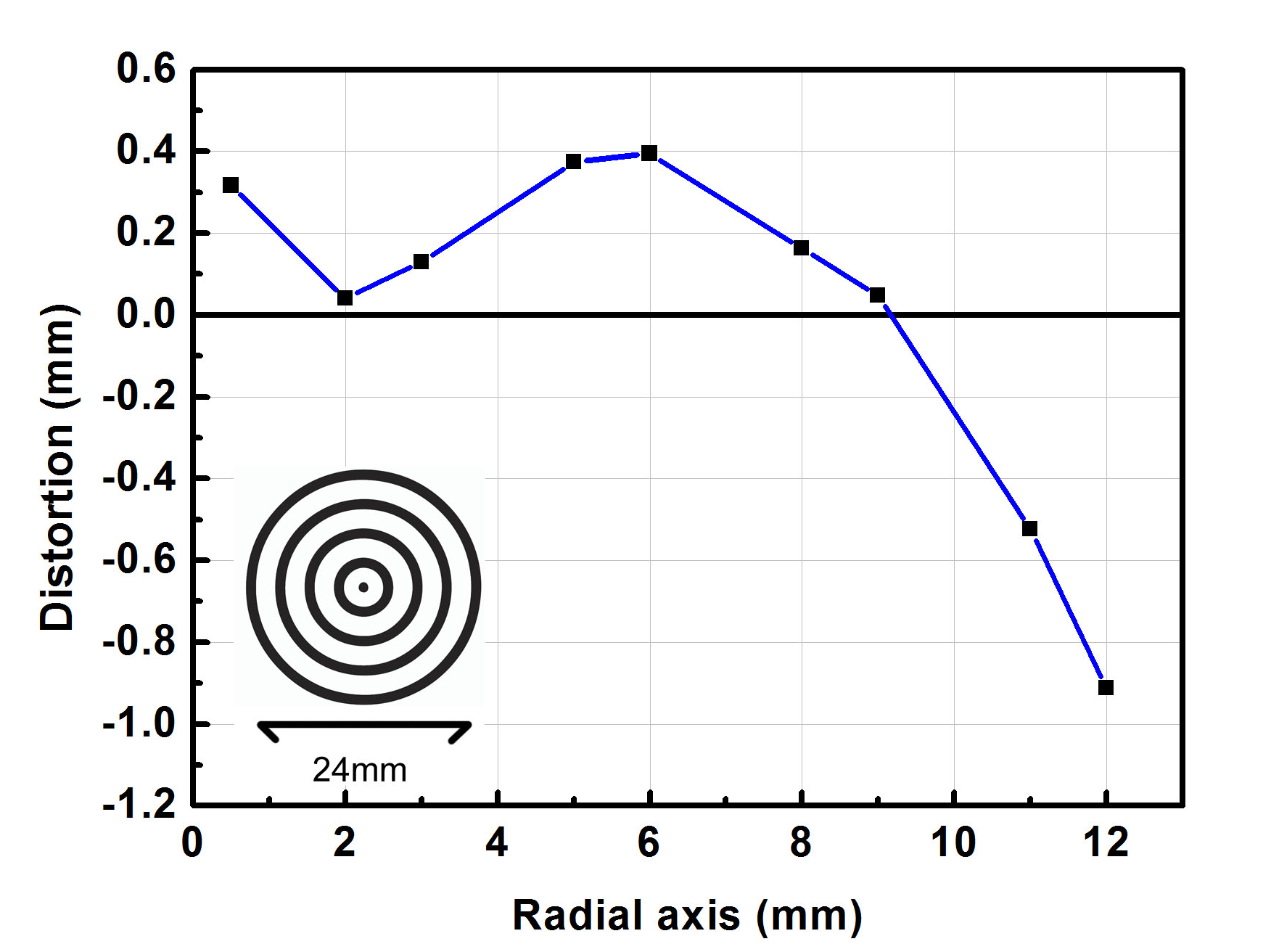


S6 Fig. Distortion from the pattern transfer process for printing on the hemispheric substrate.
