# Supplementary material for: Conformal Pad-Printing Electrically Conductive Composites onto Thermoplastic Hemispheres: Toward Sustainable Fabrication of 3-Cents Volumetric Electrically Small Antennas
Source: PLoS One. 2015 Aug 28;10(8):e0136939. doi: 10.1371/journal.pone.0136939 (PMC4552618; doi:10.1371/journal.pone.0136939)
Supplement: S7 Text — (DOC) [file pone.0136939.s007.doc]

**S7 Text. Cost estimation of the ESAs in a mass production scheme.**

The production cost of the ESAs (monthly productivity of one million pieces) was roughly calculated as follows: Six workers run two pad printing production lines in three shifts. The details about the inventory data of the ESA are listed in the next part.

S3 Table. Antenna production cost estimation.

| **Items** | **Details** | **Cost/USD** | **Cost for one piece/USD** |
| --- | --- | --- | --- |
| **Raw Materials***^a^* | PMMA base (1.2g/piece) | 0.01/piece | 0.010 |
|  | Epoxy resin (0.008 g/piece) | 6/kg | 0.000048 |
|  | Silver flakes(0.016 g/piece) | 467/kg | 0.00747 |
| **Labour***^b^* | 6 persons/month | 700/month/person | 0.0042 |
| **Energy** | 2kW/machine | 0.144/kWh | 0.000415 |
| **Depreciation of the Machines** | Two pad printing machines (3000 USD/machine) for five years’ use and consumable items | 60/month/machine | 0.00012 |
| **Workshop***^c^* | 30m^2^ | 250/month | 0.00025 |
| **Others** | - | 50/month | 0.00005 |
| **Total** | - | - | 0.022553 |

1. *We take the ESA-2 as an example for the estimation and set the costs by reference to the materials price information from Alibaba.com.*
2. *The labour cost is about the mid-range level of electronic industry in Shenzhen.*
3. *Based on the average price level in Shenzhen.*

As shown in S3 Table, when considering a mass production scenario, the total cost of a single ESA is about 2.3 cents, and the silver cost is one of the major cost drivers of the antenna. Considering the current planar RFID tag’s production cost, which involves less than 5 US cents of packaging and the chip, the overall cost of a volumetric tag based on these ESAs may rise to near 10 US cents.
