# Supplementary material for: Conformal Pad-Printing Electrically Conductive Composites onto Thermoplastic Hemispheres: Toward Sustainable Fabrication of 3-Cents Volumetric Electrically Small Antennas
Source: PLoS One. 2015 Aug 28;10(8):e0136939. doi: 10.1371/journal.pone.0136939 (PMC4552618; doi:10.1371/journal.pone.0136939)
Supplement: S8 Text — (DOC) [file pone.0136939.s008.doc]

**S8 Text. Life cycle assessment (LCA).**

1. *General information*

The present LCA was conducted according to ISO 14040 guidelines which included 4 steps: 1) goal and scope definition, 2) inventory analysis which quantifies the materials inputs, the energy inputs, and the environmental discharges through the specified life cycle phases, 3) impact assessment which accumulates flows into different impact categories, 4) interpretation of the results. ^[1]^

The system boundaries were defined as shown in S7 Fig., we simply considered about the main raw materials and the energy used in the fabrication process. In this comparison study, we considered the main environmental impacts of the antenna and the cap, and the functional unit is defined as “fabricating 10000 pieces of 3-D antennas”and “fabricating 10000 pieces of bottle caps”.

The life cycle impact assessment (LCIA) was conducted with the characterization model CML 2001-Apr.2013 incorporated in GaBi 6.0. In this assessment, we considered the most concerned environmental impact catagories: Global Warming Potential (GWP) and Photochemistry Ozone Creation Potential (POCP).


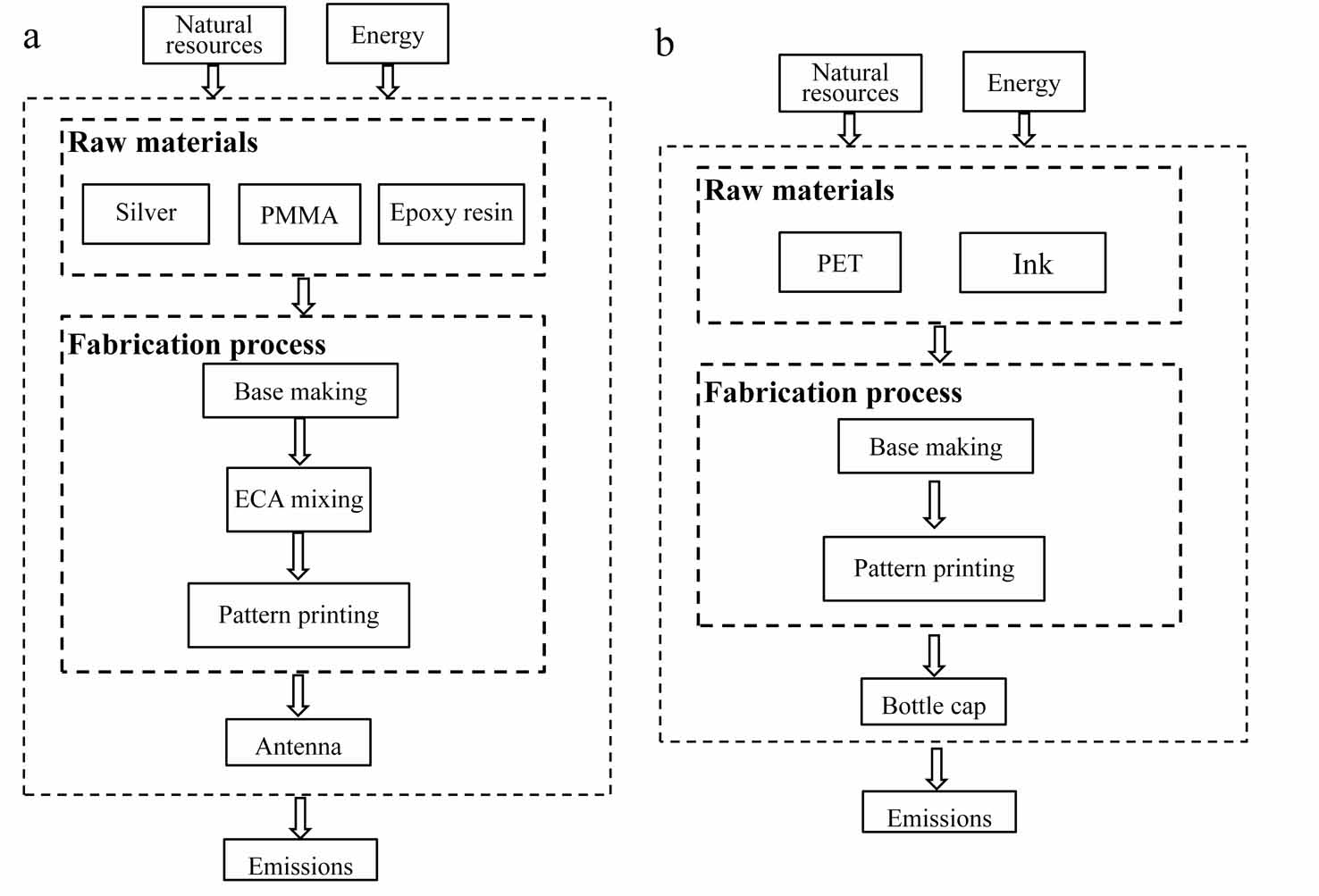


S7 Fig. System boundaries of volumetric ESA and bottle cap.

1. *Inventory data of the ESA*

The inventory data of the ESAs are calculated based on sample ESA-2, which have the largest printed area of the ECC for the three ESA samples. The weight of the PMMA hemisphere is of 1.2 g and the mass of ECC on one antenna is calculated based on the printed area (about 4.8 cm^2^ for ESA-2) and the printed thickness (about 20 μm). The density of the ECC was estimated to be 2.5 g/cm^3^. For the bottle caps, we considered the bottle cap to be made up of polypropylene (PP) resin and the mass of 2 g/piece. The energy consumption is estimated according to the set-power of the machine and the other manufacturing facilities.

1. *Life cycle impact assessment result*

We conducted a life cycle assessment (LCA) study of the antenna to quantify its environmental impacts. Since the antenna was simply composed of polymethyl methacrylate (PMMA) and ECC (epoxy resin & silver filler), we took these materials and the energy consumption in the fabrication process into consideration. The results show that the PMMA contributes the most to the impact, which is mainly because that it accounts for about 98 wt% of the antenna. In addition, although silver constitutes a small weight portion in the antenna (0.016 g/piece, 1.3 wt%), it still has considerable environmental impacts. For example, the silver contributes about 18.33% to the POCP (S8 Fig insert). This is due to the energy intensity and complex processes of mining, refining and production.

To further identify the environmental impacts of the antenna, we conducted a comparative LCA study of the ESA and a bottle cap. After standardizing the life cycle impact assessment (LCIA) results, we did a comparison as shown in S8 Fig, from which we can see that the life cycle of the volumetric ESA has similar impacts as the bottle cap does to the environment. For example, the production of 10000 caps emissions about 60.5 kg CO_2_-Equiv., while the production of 10000 pieces of antenna generates about 140 kg CO_2_-Equiv. greenhouse gas, which is just 2.31 times of the cap. The standardized LCIA results shown in S8 Fig demonstrate the similar impacts of the antenna and bottle cap in GWP and POCP. The impacts indexes of the antenna and bottle cap are of the same order of magnitude. Moreover, the higher score which antenna has is mainly due to the different processing of the raw materials.


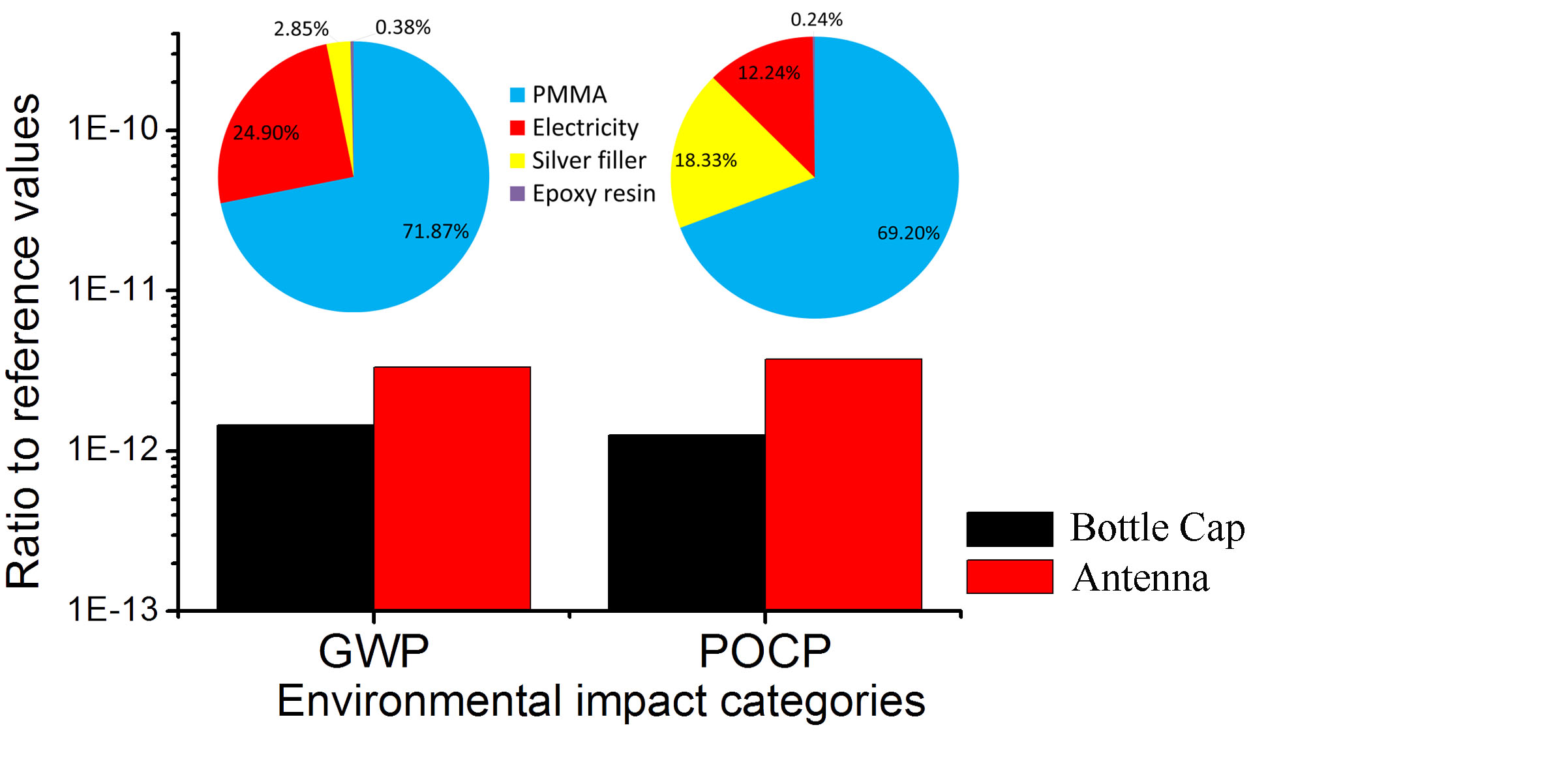


S8 Fig. Comparison of the standardized LCIA results of the ESA and the bottle cap. The insert pie charts are the contributions of the raw materials of antena to the environmental impacts of GWP (left) abd POCP (right).

**Reference**

[1] Finkbeiner M, Inaba A, Tan RBH, Christiansen K, Kluppel HJ. The New International Standards for Life Cycle Assessment: ISO 14040 and ISO 14044. *Int. J. Life Cycle Assess.* **2006**; 11(2): 80-85. doi: 10.1065/lca2006.02.002.
