# Supplementary material for: Conformal Pad-Printing Electrically Conductive Composites onto Thermoplastic Hemispheres: Toward Sustainable Fabrication of 3-Cents Volumetric Electrically Small Antennas
Source: PLoS One. 2015 Aug 28;10(8):e0136939. doi: 10.1371/journal.pone.0136939 (PMC4552618; doi:10.1371/journal.pone.0136939)
Supplement: S9 Text — (DOC) [file pone.0136939.s009.doc]

**S9 Text. Parameters used in antenna simulation.**

S4 Table. Parameters common to all three antennas used in simulation.

| **Parameters** | **Values** |
| --- | --- |
| Substrate thickness | 1.0mm |
| Substrate relative permittivity | 3.80 |
| Antenna hemisphere radius | 12.0 mm |
| ECC line thickness | 20 μm |
| ECC line roughness | 5 μm |
| ECC conductivity | 1.2 x 10^5^ S/m |

S5 Table. Specific parameters for each antenna.

| **Antenna** | Arm-count | Turn-count | ECC line width |
| --- | --- | --- | --- |
| ESA-1 | 2 arms | 2.6 turns | 1.0 mm |
| ESA-2 | 3 arms | 1.1 turns | 2.0 mm |
| ESA-3 | 4 arms | 0.9 turns | 1.5 mm |

The simulation is based on the electromagnetic finite element solver HFSS from Ansoft, Inc. The antenna model is designed according to the above parameters and then connected to an infinite ground plate, leaving the system at the center of an air-filled, cubical environment. The simulation process is then carried out until the solution converges.
